# Direct and indirect barriers to hypothetical access to care among Canadian forces health services personnel

**DOI:** 10.1007/s43999-023-00026-6

**Published:** 2023-08-02

**Authors:** Jennifer Born, Christine Frank

**Affiliations:** Department of National Defence and the Canadian Armed Forces, Director General Military Personnel Research and Analysis, Ottawa, Ontario Canada

**Keywords:** Barriers to care, Military, Mental health, Physical health, Intentions, Theoretical domains framework

## Abstract

**Background:**

Though research among Canadian Forces Health Services (CFHS) personnel is limited, the literature suggests formal healthcare is underused. Though much research has been conducted on particular barriers (e.g., stigma), examining a breadth of barriers could better inform behavioral interventions. Furthermore, work has yet to examine the indirect effects of barriers through their impact on intentions to access care.

**Methods:**

CFHS participants were randomly assigned to complete either a mental health (*N* = 503) or physical health (*N* = 530) version of the survey. The survey included questions on the perceived impact of barriers, health-related information (e.g., past access to care), intention to seek care, and two hypothetical scenarios (i.e., pneumonia and back injury or post-traumatic stress disorder and depression) as a proxy of access to care. Multiple regressions using Hayes PROCESS macro were conducted to assess the direct and indirect effects (through intentions) of the barriers on hypothetical access to care.

**Results:**

Results show conflict with career goals barriers were indirectly linked to all health outcomes, and directly linked to mental health outcomes. Treatment preference barriers were directly and indirectly linked to care seeking only for mental health, while resource barriers were directly linked to care seeking only for physical health. Knowledge and ability to access care barriers were directly linked to care seeking for depression and pneumonia.

**Implications:**

Interventions to improve treatment-seeking should be developed only after the behavioural antecedents are understood, and should focus on combining evidence-based techniques to simultaneously target multiple aspects of the behaviour.

**Supplementary Information:**

The online version contains supplementary material available at 10.1007/s43999-023-00026-6.

## Introduction

Published literature overwhelmingly suggests that military personnel under-access care when needed (e.g., [1, 2]). Studies in this area have almost exclusively focused on military members’ under-use of mental health care (e.g., [3–5]). Additionally, research examining barriers to care has heavily focused on stigma toward mental health care [6, 7]. Less is known, however, about military access to physical health care and the barriers associated with treatment-seeking for physical health issues [8, 9]. Even fewer studies explore treatment-seeking among military health care providers [10], a sub-population of military personnel at increased risk of physiological and psychological issues [11]. Current research lacks two features: (1) a theoretical understanding of the target behaviours [12], and (2) the selection of evidence-based interventions to enhance service use [1].

The Canadian Armed Forces (CAF) provides medical and dental services to their personnel in a system that is mostly independent from the publicly funded provincial health care systems. During clinic hours, CAF personnel access CAF services directly, and only access the provincial system for emergency services after hours or for specialty services not provided at their base clinic (e.g., MRI, blood tests, specialty consults). The size of the CAF health care infrastructure on a base is often proportional to the size of the base, with smaller stations providing fewer services on base, necessitating travel to a larger base nearby or outsourcing of care to provincial (or limited private) healthcare services.

### Behaviour change theory

Michie et al. [13] developed the Behaviour Change Wheel (BCW), a framework to simplify selecting interventions that are most likely to be effective. The approach links nine key intervention functions to the essential conditions that are required for behaviour (capability, motivation, and opportunity; the COM-B model [13]). The COM-B model proposes that care-seeking behaviours result from the interaction of (a) one’s motivation to access care, (b) one’s internal physical and psychological capability to act, and (c) the opportunity to do so provided by the external physical and social environments. The Theoretical Domains Framework (TDF) expands the three COM-B components into more specific theoretical domains [14, 15] that have successfully been used to explore a variety of health behaviours (e.g. [16],). Within the TDF domains, knowledge, skill, memory, attention and decision processes, and behavioural regulation fall under capability; professional role and identity, beliefs about capabilities, beliefs about consequences, nature of the behaviours, emotion, and motivation and goals fall under motivation; and social influences, environmental context and resources fall under opportunity [13]. The TDF domains can pinpoint specific behaviour change techniques (BCT) to inform theory-based interventions [13, 17].

### Intention

Another consideration when exploring access to care is the role of intention. A review by Sheeran [18] estimated that intention explained over a quarter of the variance in behaviour across a range of contexts. A meta-analysis by Webb and Sheeran [19] concluded that a change in intention generally leads to a change in behaviour, although the effect size of the change in behaviour is often smaller than that of the change in intention. Some contemporary studies have targeted increasing treatment seeking intentions in veteran and military (e.g., [20, 21]) populations by identifying and addressing barriers with varying success. Thus, research has examined the link between barriers and intention, and intention and behaviour separately. However, no research to date has examined which barriers are indirectly linked to accessing care through intention, and which are directly linked to accessing care.

### Current study

Given that the health of providers has been linked to the quality of care provided [22], in addition to the fact that military healthcare providers (HCPs) are tasked with providing high-quality care to all CAF members, the health of CAF HCPs is of particular importance. Thus, it is important to understand if CAF HCPs plan to access care and the factors that are associated with their help-seeking behaviours. Specifically, understanding the role intentions play in hypothetically accessing care will guide the development of evidence-based interventions to improve access to care for CAF HCPs.

The goal of the current research is (1) to assess hypothetical care seeking (using health scenarios) for mental and physical health issues among CFHS personnel and (2) to determine if the relationships between perceived barriers and hypothetical care seeking (for both mental and physical health issues) are direct associations (i.e., a barrier acts directly on the hypothetical behaviour) or indirect associations (i.e., a barrier impacts hypothetical behaviour through intention to seek care).

## Methods

### Participants and procedures

In May 2019, all CFHS personnel with a valid email (*N* = 3,171) were invited to participate in a study examining barriers to care. Written informed consent (recorded electronically) was obtained from all subjects. Consenting participants were randomly assigned to complete either a survey assessing hypothetical access to care for mental health issues or a survey assessing hypothetical access to care for physical health issues. The two versions of the survey were identical aside from the scenarios and a single item assessing intention to access care, which specified whether intentions were related to accessing mental or physical care. In addition, participants were shown the following prompt prior to responding to the barrier items: “The following statements relate to seeking care for *physical health [or mental health]* issues exclusively.” All procedures contributing to this work comply with the ethical standards of the relevant national and institutional committees on human experimentation and with the Helsinki Declaration of 1975, as revised in 2008. This study was approved by the Department of National Defence / Canadian Armed Forces Social Science Research Review Board (#1801/18F).

A review of the patterns of missing data identified several records that contained responses to very few items. These empty records (i.e., missing more than 50% of items from each section) were excluded from the analysis. Data from 1,033 participants (32.6% response rate) were included in the analysis. Five hundred and thirty individuals completed the physical health version of the survey, and 503 completed the mental health version of the survey. The majority of respondents (53.4%) were CAF members employed in core clinical trades (i.e., nursing officers, medical officer’s physician assistants, or medical technicians), were junior non-commissioned members (28.8%) or junior officers (31.1%), between 30 and 49 years of age (67.8%), were English speakers (82.1%), and lived in an urban setting (60.8%). The sample presented comparable proportions between male (46.1%) and female (47.9%) respondents. See Online Supplement 1 for description of samples.

### Measures

#### Covariates

In addition to gender, age, rank, trade, language, and location^1^; past negative experiences with care, past diagnosis, past year access to care, and self-reported health were considered potential covariates (see [23] for details). Negative past experiences were assessed with a single item that asked participants to what extent they agreed with the following statement on a 6-point scale (ranging from *Strongly Disagree* to *Strongly Agree*), “I have had past negative experiences when accessing care” (*M*_*PHYSICAL*_ = 2.80, *SD* = 1.61; *M*_*MENTAL*_ = 2.81*, SD* = 1.66).

Whether participants had received a mental or physical health diagnosis in the past two years was assessed by asking “Have you been diagnosed with a chronic injury or had a serious injury in the past 2 years?” and “Have you been diagnosed with a mental health disorder in the past 2 years?” Participants were coded as having no past diagnosis, having received a physical health diagnosis, having received a mental health diagnosis, or having received both a mental health and physical health diagnosis.

Past year access to care was assessed by asking participants to report actual use of formal services using the item “How many times in the past year have you sought formal care for an illness?” Due to extreme skew, the variable was dichotomized, with zero responses coded as zero and any response above zero coded as one.

Last, self-rated health was assessed with a single item asking respondents “In general, how would you rate your *physical* [*mental*] health?” with respondents indicating very poor, poor, fair, very good, or excellent.

#### Barriers

Barriers to care were assessed with the Barriers to Care Scale [24]. The scale was developed using the TDF [14, 15] and included 46 items mapping onto eight barrier factors under the COM-B model, including: capability—(1) Knowledge and ability to access care (e.g., I find it difficult to navigate the administrative processes necessary to seek some types of care [e.g., mental health, civilian care]); opportunity—(2) Staffing and workload resources (e.g., I would have difficulty getting time off to access care); (3) Organizational and social support (e.g., My immediate supervisor does not support my accessing health services); and motivations (4) CAF healthcare provider identity (e.g., If I accessed care, members of my unit might have less confidence in me as a health care provider); (5) Discomfort accessing care at work (e.g., I’m uncomfortable receiving care from colleagues); (6) Conflicts with career goals (e.g., Accessing care will harm my future chances of promotion); (7) Concerns about confidentiality (e.g., When I access care, my colleagues are able to see why I’ve sought care in the past); and (8) Treatment preferences (e.g., I want to solve the problem on my own rather than access care). Participants were asked to indicate to what degree the following barrier would prevent them from accessing care on a 6-point scale (ranging from *Extremely unlikely* to *Extremely likely*; see Online Supplement 2 for a list of barriers and survey items and details about the scoring of barrier factors.). The barriers scale had good reliability, with subscale reliabilities ranging from α = 0.86 to 0.97. Mean scores for each barrier subscale were computed, with higher scores indicating a higher perceived impact.

Mean barrier scores in the physical health sample ranged from 1.85 (for Organizational and social support) to 3.40 (for Staffing and workload resources), while scores in the mental health sample ranged from 1.97 (for Organizational and social support) to 3.69 (for Discomfort accessing care at work, see Table 1).

**Table 1:** Mean barrier factor scores within mental and physical health samples

Component	Factor	X	Physical Health		Mental Health	
			*M*	*SD*	*M*	*SD*
Capability	Knowledge and ability to access care	X_3_	2.17	1.14	2.31	1.16
Opportunity	Staffing and workload resources	X_4_	3.40	1.37	3.37	1.39
	Organizational and social support	X_6_	1.85	1.10	1.97	1.07
Motivation	CFHS personnel identity	X_1_	2.53	1.24	2.93	1.37
	Discomfort accessing care at work	X_2_	3.38	1.44	3.69	1.55
	Conflicts with career goals	X_5_	3.20	1.31	3.49	1.31
	Treatment preferences	X_8_	3.39	1.15	3.39	1.17
	Concerns about privacy	X_7_	2.82	1.42	2.94	1.46

#### Intention

Intention to access health care services was assessed using a single question: “When faced with a *physical* [*or mental*] health issue, I intend to access care.” Participants indicated their level of agreement on a 7-point scale (from 1 *Strongly Disagree* to 7 S*trongly Agree*). Mean intention scores were 4.75 (*SD* = 1.69) for mental health and 5.27 (*SD* = 1.60) for physical health.

#### Hypothetical access to care

Hypothetical access to care was assessed using two vignettes for each version of the survey: pneumonia and back injury for the physical health version, and posttraumatic stress disorder (PTSD) and depression for the mental health version (See Online Supplement 2 for health scenarios). The scenarios each contained four steps, with step-1 presenting mild symptoms, and each subsequent step increasing in symptom and illness severity. The progression was such that at step-1 self-treatment was appropriate; at step-2 accessing formalized care would be reasonable; and at step-3 accessing formalized care would be the only appropriate action. At step-4 accessing formalized care was still the only appropriate action, but this scenario presented more severe symptoms than in step-3. For the current study, the outcomes will be measured at the third step (step-3) of each scenario because this was the first point in the 4-step scenario when accessing formalized care was the only appropriate option. For each step of the scenario, participants were presented with five choices: (a) I would do nothing/wait and see; (b) I would self-treat; (c) I would informally consult a colleague or peer; (d) I would seek formal treatment using CAF health services; and (e) I would seek formal treatment using civilian health services. Responses to step-3 of the scenarios were dichotomized where the response options indicating formalized treatment using either CAF or civilian health services were coded as one and all other options were coded as zero. See Online Supplement 2 for a list of scenario survey items and the scoring of hypothetical access to care.

### Analysis

Secondary analysis of survey data included three steps. First, prior to testing the direct and indirect effects, and to avoid overfitting the model, covariates were tested for inclusion in the model. Univariate ANOVAs were examined to assess the associations between hypothetical access to care for each of the four scenarios and gender, age, rank, trade, language, location, past negative experiences with care, past diagnosis, past year access to care, and self-rated health. Only covariates that had a significant association with hypothetical access to care (scenario-specific) were included in the final model.

Second, prior to calculating the direct and indirect effects, multi-collinearity was assessed by entering the barrier factors and intention to access care into a regression, predicting hypothetical access to care for pneumonia as the outcome to generate Variance Inflation Factors (VIFs). The VIFs ranged between 1.21 and 3.23, indicating no issues with multi-collinearity.

Last, while controlling for the relevant covariates, the eight barrier factors were included as predictors (X_1_-X_8_), with intention to access care as the mediator (M), and hypothetical access to care as the dichotomous outcome variable (Y). The direct effects of each barrier factor on hypothetical access to care, while controlling for the other variables (i.e., other barrier factors, relevant covariates, and intentions to access care), and indirect effects of each barrier factor on hypothetical access to care through intentions (while controlling for other barrier factors and relevant covariates) were computed using Hayes (2009) PROCESS macro [25]. Direct and indirect effects are estimated using ordinary least squares (OLS) regression analyses separately for each barrier factor with bootstrapping (5000 replications). PROCESS has been found to produce more reliable estimates [26] than the causal step method by Baron and Kenny [27] or the also commonly used Sobel test [28].

## Results

The majority of each sample (83.1% of respondents in the physical health sample, and 74.9% in the mental health sample) reported that they had actually accessed care at least once in the past year. Less than half of respondents indicated they would seek formal treatment when appropriate in the health scenarios. At the first step, when symptoms dictated that accessing formalized care would be the only appropriate action, only 38.2% would hypothetically seek care for pneumonia, 42.0% for a back injury, 25.9% for depression, and 34.6% for PTSD.

The associations between the covariates (gender, age, rank, trade, language, location, past negative experiences with care, past diagnosis, past year access to care, and perceived health) and hypothetical access to care across the four scenarios were assessed. Perceived health, *F*(4, 1025) = 9.81, *p* < 0.001, past negative experiences, *F*(5, 960) = 2.46, *p* = 0.03, and past year access to care, *F*(1, 1024) = 16.74, *p* < 0.001, were significantly associated with hypothetical access to care for back injury. Perceived health, *F*(4, 1025) = 6.33, *p* < 0.001, past negative experiences, *F*(5, 960) = 4.11, *p* = 0.001, and past year access to care, *F*(1, 1024) = 18.47, *p* < 0.001, were significantly associated with hypothetical access to care for pneumonia. Past negative experiences, *F*(5, 960) = 7.20, *p* < 0.001, were significantly associated with hypothetical access to care for depression. Past negative experiences, *F*(5, 960) = 4.78, *p* < 0.001, and past year access to care, *F*(1, 1024) = 6.03, *p* = 0.01, were significantly associated with hypothetical access to care for PTSD.

Tables 2 summarises the direct and indirect effects (via intention) of barriers for each scenario; only the direct and indirect effects (i.e., the unstandardized regression coefficients and standard errors) will be highlighted below. Significant results are bolded. The complete results of the direct and indirect results of the regression analyses are included in Online Supplement 3.

**Table 2:** Summary of full direct and indirect effects for barriers to care seeking scenarios

Component	Factor (Model Parameter)	Physical health				Mental health			
		Pneumonia		Back injury		Depression		PTSD	
		DE	IE	DE	IE	DE	IE	DE	IE
		*B(se)*	*B(se)*	*B(se)*	*B(se)*	*B(se)*	*B(se)*	*B(se)*	*B(se)*
**Capability**	Knowledge and ability to access care (X_3_)	**-.30(.13)**	-.02(.03)	.01(.13)	-.02(.03)	**-.37(.14)**	-.04(.03)	.01(.13)	-.03(.03)
**Opportunity**	Staffing and workload resources (X_4_)	**-.37(.14)**	-.05(.03)	-.50(.15)	-.04(.03)	-.20(.13)	.02(.03)	-.10(.14)	.01(.03)
	Organizational and social support (X_6_)	.09(.17)	.02(.04)	-.22(.17)	.03(.04)	-.01(.17)	.01(.04)	-.19(.16)	.01(.04)
**Motivation**	CFHS personnel identity (X_1_)	.11(.20)	-.07(.05)	.38(.21)	-.08(.05)	.28(.19)	-.01(.05)	.16(.20)	-.02(.05)
	Discomfort accessing care at work (X_2_)	-.15(.15)	.03(.03)	-.04(.17)	.04(.03)	-.13(.15)	-.01(.03)	.18(.16)	.01(.03)
	Conflicts with career goals (X_5_)	-.04(.15)	**-.07(.04)**	-.01(.16)	**-.08(.04)**	**-.35(.15)**	**-.08(.04)**	**-.46(.17)**	**-.08(.04)**
	Treatment preferences (X_8_)	-.19(.16)	-.01(.03)	-.05(.17)	-.03(.03)	**-.47(.17)**	**-.08(.05)**	**-.52(.18)**	**-.10(.05)**
	Concerns about privacy (X_7_)	.28(.15)	.01(.03)	-.19(.15)	.02(.03)	.12(.16)	.02(.04)	-.13(.16)	.01(.03)
**Intention**	(M)	**.27(06)**		**.33(.09)**		**.30(.08)**		**.29(.08)**	

*DE* Direct Effects, *IE* Indirect Effects, *B* – Coefficient, *se* – Standard error, Significant results at *p* < .05 are in **bold**

### Back injury and pneumonia

Conflicts with career goals was *indirectly* linked to hypothetical care seeking for both back injury and pneumonia. Staffing and workload resources was *directly* linked to hypothetical care seeking for both physical health scenarios. Knowledge and ability to access care was *directly* linked to hypothetical care seeking for pneumonia.

### Depression and PTSD

Conflicts with career goals and treatment preferences were both *directly* and *indirectly* linked to hypothetical care seeking for both depression and PTSD. Additionally, knowledge and ability to access care was *directly* linked to hypothetical care seeking for depression.

## Discussion

### Main findings

To our knowledge this is the first study to assess the direct and indirect impact of barrier factors on hypothetical access to care for both mental and physical health issues in a provider or military population. First and foremost, the percentage of those who indicated they would access care in the vignettes suggests that CFHS personnel may under-access care in real-world settings. Though accessing formalized care was the only appropriate choice at step-3 of the hypothetical health scenarios, less than half of respondents indicated that they would access care for the physical health issues (38–42%) and approximately a third or less indicated they would access care for the mental health issues (26–35%). In line with past research, hypothetical access to care for mental health issues was low (e.g., [29]).

Second, supporting past findings [18], intention was a consistent and strong predictor of hypothetical behaviour. In our models, intention had a significant and direct effect on hypothetical access to care in all four scenarios (pneumonia, back injury, depression, and PTSD). When examining the standardized scores (i.e., Z-scores) of all the predictors in the models, intention consistently had the strongest association with hypothetical access to care.

Finally, the results of the present study indicate that the effect of barriers on treatment-seeking in CFHS personnel differs based on context. Where Treatment preferences (motivation) was directly linked to treatment-seeking for only mental health issues, Staffing and workload resources (opportunity) was directly linked to treatment-seeking only for physical health issues. Additionally, Knowledge and ability to access care (capability) was directly linked to treatment-seeking for pneumonia and depression. The effect of Conflict with career goals (motivation) was consistent across scenarios, directly and indirectly linked to treatment-seeking for both mental health scenario outcomes, and indirectly linked to treatment-seeking for both a back injury and pneumonia.

### Interpretation of results

This study was guided by the COM-B model and the TDF. From a theoretical perspective, this study has confirmed that barriers related to capability (i.e., Knowledge and ability to access care) and physical opportunity (i.e., Staffing and workload resources) acted directly on hypothetical behaviour (independent from intentions), suggesting these barriers are likely post-intentional. For example, an individual may have a high intention to access care when needed, but then they are short-staffed at work and must make a post-intentional decision whether to access care. Only barriers that mapped to domains related to motivation (i.e., Treatment preferences and Conflict with career goals) were indirectly associated with hypothetical behaviour (i.e., mediated through intention).

Theory suggests that staffing and workload issues are an opportunity barrier (more specifically, physical opportunity), which the BCW [13] advises could be addressed through environmental restructuring (e.g., changing the system to normalize access to care from providers outside the chain of command), enablement (e.g., providing time off to access care), or restriction (e.g., making rules to increase access to care by reducing the opportunity to engage in competing behaviours, namely rules against self-treatment and hallway consultations). A lack of knowledge and ability to access care falls under capability (more specifically, psychological capability) and can be effectively targeted through training (e.g., imparting skills to navigate the health care system).

Conflicts with career goals, Treatment preference and intention fall under motivation in the COM-B model. BCW suggests several broad intervention functions, including education, persuasion, incentivization, and coercion, for reflective and automatic motivational processes [13]. More familiar education-based interventions are poised to have a greater impact on improving access to care through reflective processes, and thus should avoid targeting the automatic or impulsive aspects of accessing care, such as emotions [13]. Instead, education could provide information on changes in policies and procedures for accessing care and clarify the impact of accessing care on promotions and career progression. Exploring these barriers at the TDF domain level provides additional details on potential interventions. For example, Conflicts with career goals would be mapped under goals in the validated TDF [15], as these barriers reflect theoretical constructs related to goal setting and goal priorities. Goal setting (example in this context: formulating clear goals to access care when short on time), and behavioural contracts (example in this context: signing a contract with your military supervisor agreeing that you will access care when needed during the next calendar year) are two types of BTC that could address behavioural regulation and goals, respectively [17].

The results (in Table 2) suggest that physical opportunity barriers only have a direct impact on access to care behaviours in physical health contexts. Understanding whether barriers act through or are independent of intention is important for the development and implementation of successful interventions. As encouraged by Michie [30], interventions that act simultaneously to target different aspects of the behaviour will be more effective at changing the behaviour in the short and long terms. Interventions that only target one type of barrier may have reduced effectiveness at changing behaviour, particularly those that target post-intentional barriers in populations with low intentions to access care.

From this point, the theoretical understanding of barriers faced by CFHS personnel can inform the development of intervention strategies. Methods similar to those employed by Michie et al. [31], can be used to develop a comprehensive list of behavioural change techniques for each barrier. Combining techniques to address multiple types of barriers would produce a complex but comprehensive theory-based behavioural change intervention to improve access to care for CFHS personnel. In order to systematically build on the knowledge base, the next steps involve implementing and assessing the effectiveness of these interventions. Trials should be purposefully designed to measure the relative impact of each BTC while ensuring to note the specific context and population.

### Limitations

As with most survey research, all of our measures (including the hypothetical behavioural outcome) were self-reported, introducing several potential sources of bias. We anticipate that the direction of the biases may be similar for all subjective measures and thus may have inflated the strength of association in this study, particularly between self-reported intention and the self-reported behavioural outcomes.

Additionally, approximately a third of CAF HCPs responded to the survey. The limited response rate may have resulted in some biases. For example, HCPs who struggle with resources, such as a lack of time to seek care, may also struggle with finding the time to respond to a survey. As a result, the findings are not necessarily generalizable to all CAF HCPs. Additional research is needed to confirm if these relationships hold in other populations, such as civilian HCPs and the wider CAF.

Though it is common practice to use vignettes in behavioural studies, scenarios can only provide a proxy for behaviour [32]. The use of vignettes allowed us to capture hypothetical care data in a variety of contexts not easily obtainable using other methods. Yet, it is not possible to accurately capture the reality faced by CAF HCPs using a brief survey-based scenario [33]. Future studies should expand on these findings to assess the relationship between barriers and intention and measures of actual care seeking propensity, such as healthcare visits for mental and physical health problems. However, it is worth noting that the percent of those who accessed formal care was still relatively low, indicating that many respondents likely were not influenced by social desirability bias in this particular assessment. Additionally, The use of vignettes and the breadth of barrier factors was also a strength of the study as it allowed us to highlight significant differences across a variety of conditions rather than focus on a specific health condition. Studies that are focused on one particular barrier type (e.g., stigma) or context (e.g., access to physical health) may miss critical opportunities to identify a range of issues related to access to care.

A third limitation is that we included CFHS personnel in the analyses who did not currently report a health problem. The literature suggests that the perception of barriers by individuals needing or in treatment may be very different from that of those facing a hypothetical problem [6, 21]. We did control for past diagnosis; however, in order to better mitigate this limitation, longitudinal studies are needed to explore the causal relationships between health status, barrier perceptions, and treatment seeking.

Last, the extent to which the military and medical contexts influence the perception of barriers remains unclear. In the military, even minor health issues can disrupt a career through a failure to be cleared medically at a key point in one’s progression (i.e., before a mission or course). While healthcare providers are also reluctant to report health issues [34], all too aware that a mental health diagnosis could threaten their license to practice, military providers may be even more motivated to self-treat, as both military [8] and medical cultures [35] value self-reliance and performance. Though our results were generally comparable to other findings in military and provider populations, without a common measure or a methodological consensus on how best to measure barriers [6], additional comparisons between studies to provide insight into the contexts (e.g., when and for whom access to care is more difficult) are not yet possible.

## Conclusion

This study has broadened our understanding of the relationships between barriers, intentions, and hypothetical treatment-seeking. Mental health and physical health scenarios were associated with some similar types of barriers but also revealed some context-specific findings. It is important to note that our findings suggest that barrier reduction strategies may not be effective across all contexts (e.g., they may only be effective for certain types of mental health issues or for physical health issues but not mental health issues). Recognizing the context in which the barriers exercise the most influence is crucial when creating and implementing interventions. Interventions to improve treatment-seeking should be developed only after the behavioural antecedents are understood, and they should focus on combining evidence-based techniques to simultaneously target multiple aspects of the behaviour. Resources and knowledge barriers may be directly addressed to attenuate their effects, while career goals and treatment preference barriers can be weakened not only directly, but also indirectly by targeting the intention to access care. Uniquely, this study has quantified the association between barriers, intentions, and hypothetical treatment-seeking in a variety of health contexts (including pneumonia, back pain, PTSD, and depression), emphasizing the importance of direct and indirect effects through intentions in these relationships. Future work should continue to explore these associations in the wider military and health care provider populations, using a congruent set of barriers and a variety of health contexts. Ideally, future studies in military populations should assess the direct and indirect impact of theoretical barriers on actual care-seeking behaviours while additionally controlling for current health status.

### Supplementary Information

**Additional file 1.** 

**Additional file 2.** 

**Additional file 3.**

## Data Availability

The data are not publicly available due to information that could compromise the privacy of research participants. The data that support the findings of this study can be requested electronically by completing an access to information request at https://atip-aiprp.apps.gc.ca/atip/privacyTerms.do (see https://www.canada.ca/en/department-national-defence/corporate/transparency/access-information-privacy.html for details).

## Footnotes

1 Location was based on their posting location and condensed into three categories using Statistics Canada’s guidelines (i.e., rural, peri-urban, and urban). The rurality of the respondent’s location was included as a covariate as it may impact the type of and quality of health care services locally available and the travel burdens to access more specialized services.
