# Correction: Direct and indirect barriers to hypothetical access to care among Canadian forces health services personnel

**DOI:** 10.1007/s43999-023-00035-5

**Published:** 2023-12-08

**Authors:** Jennifer Born, Christine Frank

**Affiliations:** Department of National Defence and the Canadian Armed Forces, Director General Military Personnel Research and Analysis, Ottawa, Ontario Canada

**Correction: Res Health Services Regions 2: 11 (2023)**

**https://doi.org/10.1007/s43999-023-00026-6**

Following the publication of the original article [1], the authors identified that the copyright line was incorrect. The correct copyright line is given below.

The incorrect copyright line is:

© Crown 2023

The correct copyright line is:

© His Majesty the King in Right of Canada 2023

The original article [1] has been corrected.

## References

[1] Born J, Frank C (2023) Direct and indirect barriers to hypothetical access to care among Canadian forces health services. Res Health Services Regions 2:11. 10.1007/s43999-023-00026-6 10.1007/s43999-023-00026-6 PMC11281741 39177892

