# Supplementary material for: Direct and indirect barriers to hypothetical access to care among Canadian forces health services personnel
Source: Res Health Serv Reg. 2023 Aug 2;2:11. doi: 10.1007/s43999-023-00026-6 (PMC11281741; doi:10.1007/s43999-023-00026-6)
Supplement: Supplementary file 1 — Additional file 1. [file 43999_2023_26_MOESM1_ESM.docx]

**Online Supplement 1 - Sample descriptions**

Table 1.1

*Demographic and health information of the survey sample*

|  |  | Physical Health  *n* = 530 | Mental Health  *n* = 503 | All  *n* = 1033 |
| --- | --- | --- | --- | --- |
| Variable | Categories | % | % | % |
| Gender | Male | 45.7% | 46.5% | 46.1% |
|  | Female | 48.9% | 46.9% | 47.9% |
|  | Not reported | 5.5% | 6.6% | 6.0% |
| Age | 20-29 | 17.0% | 16.3% | 16.7% |
|  | 30-39 | 37.0% | 37.0% | 37.0% |
|  | 40-49 | 30.8% | 26.0% | 28.5% |
|  | 50 and older | 11.1% | 15.3% | 13.2% |
|  | Not reported | 4.2% | 5.4% | 4.7% |
| Rank | Non-commissioned Member | 27.9% | 29.6% | 28.8% |
|  | Non-commissioned Officer | 16.6% | 16.1% | 16.4% |
|  | Junior Officer | 33.0% | 29.0% | 31.1% |
|  | Senior Officer | 18.1% | 18.5% | 18.3% |
|  | Not reported | 4.3% | 6.8% | 5.5% |
| Trade | Core Clinic | 56.2% | 50.5% | 53.4% |
|  | Close support | 18.9% | 18.3% | 18.6% |
|  | Speciality | 8.1% | 10.9% | 9.5% |
|  | Dental | 10.8% | 9.7% | 10.3% |
|  | Not reported | 6.0% | 10.5% | 8.2% |
| Preferred language for accessing care | English | 82.3% | 81.9% | 82.1% |
|  | French | 14.2% | 12.7% | 13.5% |
|  | Not reported | 3.6% | 5.4% | 4.5% |
| Location at time of survey | Rural | 2.3% | 4.4% | 3.3% |
|  | Semi-rural | 22.3% | 23.6% | 22.9% |
|  | Peri-urban | 14.6% | 16.0% | 15.2% |
|  | Urban | 60.8% | 56.0% | 58.5% |
| Past Diagnosis | None | 63.9% | 62.1% | - |
|  | Physical health diagnosis | 18.1% | 18.3% | - |
|  | Mental health diagnosis | 8.5% | 10.9% | - |
|  | Mental and physical health diagnoses | 9.5% | 8.7% | - |
|  | None | 63.9% | 62.1% | - |
| Self-rates health | Very Poor | 0.6% | 1.8% | - |
|  | Poor | 4.7% | 12.5% | - |
|  | Fair | 32.3% | 37.0% | - |
|  | Very Good | 49.1% | 38.2% | - |
|  | Excellent | 13.0% | 10.3% | - |
