# Supplementary material for: Direct and indirect barriers to hypothetical access to care among Canadian forces health services personnel
Source: Res Health Serv Reg. 2023 Aug 2;2:11. doi: 10.1007/s43999-023-00026-6 (PMC11281741; doi:10.1007/s43999-023-00026-6)
Supplement: Supplementary file 2 — Additional file 2. [file 43999_2023_26_MOESM2_ESM.docx]

**Online Supplement 2 – Survey items and scoring of responses options.**

1. Outcomes – Hypothetical access to care using four health scenarios with increasing symptoms severity.

| **Scenario** | **Scenario wording** - In this next section, you are asked to read the scenarios and indicate which action you would be most likely to take given the information and your current situation.  Option for each step in each scenario below were:   1. I would do nothing/I would wait and see 2. I would self-treat 3. I would informally consult a colleague or peer 4. I would seek formal treatment using CAF health services 5. I would seek formal treatment using civilian health services (bypass CAF services) | **Scoring** |
| --- | --- | --- |
| Pneumonia | **Step 1 -** You wake up in the morning and notice that you have a sore throat and a slight non-productive cough. The symptoms persist through the day and have not resolved when you wake up the following day.  **Step 2** - By the second day, your symptoms continue and now your cough has become productive and you are developing a low grade fever. You’ve also noticed that your breathing is more laboured.  **Step 3 -** Your cough has progressed (discoloured sputum) and worsened. You continue to experience a fever and you are now also experiencing chills and muscle pains. You also hear a crackling sound when you inhale/exhale.  **Step 4** - You still have a fever and now you’re also vomiting. You still have a productive cough with laboured breathing accompanied by a crackling sound. | Respondents who selected options d. or e. at **Step 3** scored as 1 = hypothetically access healthcare; while those selected options a., b., or c.. at **Step 3** scored as 0 = did not hypothetically access healthcare. |

| **Scenario** | **Scenario wording** - In this next section, you are asked to read the scenarios and indicate which action you would be most likely to take given the information and your current situation.  Option for each step in each scenario below were:   1. I would do nothing/I would wait and see 2. I would self-treat 3. I would informally consult a colleague or peer 4. I would seek formal treatment using CAF health services 5. I would seek formal treatment using civilian health services (bypass CAF services) | **Scoring** |
| --- | --- | --- |
| Back injury | **Step 1** - While helping to mobilize a patient, you experience some discomfort in your lower back. Later that day, the discomfort is still present with the pain increasing and decreasing throughout the day.  **Step 2** - A week later, the pain is still present and, though it does come and go, it is generally persistent. You experience pain if you sit or stand in the same position for long periods of time. Your sleep has also been disrupted because of the pain.  **Step 3** - It’s now been two months since the injury and you’re still experiencing pain. Some days it’s worse than others. You’re unable to sit or stand for long periods of time without pain.  **Step 4** - Six months have passed and the back pain comes and goes. Some days it’s better than others. You find your ability for normal daily activity to be reduced and you’re unable to perform your duties without discomfort. | Respondents who selected options d. or e. at **Step 3** scored as 1 = hypothetically access healthcare; while those selected options a., b., or c.. at **Step 3** scored as 0 = did not hypothetically access healthcare. |

| **Scenario** | **Scenario wording** - In this next section, you are asked to read the scenarios and indicate which action you would be most likely to take given the information and your current situation.  Option for each step in each scenario below were:   1. I would do nothing/I would wait and see 2. I would self-treat 3. I would informally consult a colleague or peer 4. I would seek formal treatment using CAF health services 5. I would seek formal treatment using civilian health services (bypass CAF services) | **Scoring** |
| --- | --- | --- |
| Depression | **Step 1** - Work has been stressful lately and you find yourself with less energy than usual. You feel like your work performance is really slipping lately and you’re having trouble concentrating on your tasks. You don’t find that work is as enjoyable or interesting as you usually do. You’re still engaging in typical activities at home, but because of exhaustion, it takes more effort than it normally does.  **Step 2** - A week has passed and work has settled down; however, you still find your energy affected. A few colleagues have noted that you’re finishing tasks more slowly than you normally do, and you feel guilty that you’re letting them down. You’re still having trouble concentrating at work and feel indecisive when asked to weigh in. Work just isn’t enjoyable anymore, and when you go home, you feel tired and a bit empty. You’ve started to do less around the house (e.g., doing fewer chores around the house) in favour of getting into bed earlier.    **Step 3** - It’s been two weeks, but you haven’t returned to normal. You’ve been taking sick days but, when you do go to work, you feel useless and that you’re more of a burden than a help. You still don’t have any energy and it isn’t helped by the fact you’re having trouble sleeping at night and fighting the urge to sleep during the day. Throughout most of the day, you find yourself feeling sad or empty. At home, you’re mostly inactive, laying on the couch watching TV. You don’t have the drive or interest to do anything else.  **Step 4** - It’s been about a month and you think you’re getting worse. Your mood, for one, has worsened to the point where you feel depressed and hopeless almost all of the time. You’re not finding pleasure in any of the things you did before. Work is no longer interesting. Your sleep is still disturbed. You’re still going to work, but your performance is poor by your own estimation, and it still takes you longer to complete tasks that you used to be able to do in half the time. Your focus is off and you’re just moving slower. You’re not sure of the point of you being there at all. You’ve stopped going out because it’s too exhausting and overwhelming, so you continue spending a lot of time alone. | Respondents who selected options d. or e. at **Step 3** scored as 1 = hypothetically access healthcare; while those selected options a., b., or c.. at **Step 3** scored as 0 = did not hypothetically access healthcare. |

| **Scenario** | **Scenario wording** - In this next section, you are asked to read the scenarios and indicate which action you would be most likely to take given the information and your current situation.  Option for each step in each scenario below were:   1. I would do nothing/I would wait and see 2. I would self-treat 3. I would informally consult a colleague or peer 4. I would seek formal treatment using CAF health services 5. I would seek formal treatment using civilian health services (bypass CAF services) | **Scoring** |
| --- | --- | --- |
| Post traumatic stress disorder | **Step 1** - A week ago, you returned from a disaster relief effort in a densely populated area that resulted in many casualties. It was a highly demanding deployment and you’re still thinking about your experience while you were over there. You’ve noticed yourself becoming more tense than usual. There doesn’t seem to be a reason for your tension and yet it continues to persist, making you a bit more irritable than normal.    **Step 2** - Another week passes and you continue to feel tense and irritable. You also feel hyperaware of everything around you which makes it hard for you to concentrate. When your tenseness does calm down, you find yourself feeling nothing, almost like a numbness. You’re less interested in work and you find your ability to concentrate diminishing. Being around others is more grating than usual, you find yourself feeling angry and annoyed at little things.    **Step 3** - It’s been over a month, and you find yourself thinking about your relief efforts and picturing the devastation you saw. When people ask you about your deployment, you tell them that you’d rather not discuss it. In fact, you find yourself pushing away or detaching from friends and family. With your alternating tenseness and numbness continuing, you’re finding it hard to connect with other people anyways. Plus, you’re agitated a lot of the time and you become irritated easily. You’ve started to experience insomnia and, when you do sleep, you have nightmares.    **Step 4** - It’s been about three to six months, you’re still irritable, and lately that has manifested in angry outbursts over trivial matters. You continue to feel detached from others including your closest family and friends. In between feelings of anger, you feel nothing, and find yourself unable to enjoy your hobbies. You continue to have intrusive thoughts where you think about your disaster relief experience and you find you’ve started having nightmares about it. You’ve also found that crowded spaces make you feel tense and you now avoid going to places where there might be large crowds. You realize that, based on what you saw, you think that generally people can’t be trusted. | Respondents who selected options d. or e. at **Step 3** scored as 1 = hypothetically access healthcare; while those selected options a., b., or c.. at **Step 3** scored as 0 = did not hypothetically access healthcare. |

1. Barriers – Self-rated impact of barrier factor on accessing either physical or mental health care.

| **Factor** | **Barrier wording** - The following statements relate to seeking care for [*mental health* or *physical health*] issues exclusively. Accessing care in this survey always refers to formalized care using the health services system (e.g., through sick parade or an appointment).  To what extent would this prevent you from seeking care? | **Scoring** |
| --- | --- | --- |
| CFHS personnel identity (X_1_) | If I accessed care, members of my unit might have less confidence in me as a health care provider.  Seeking care may undermine my authority in the eyes of my subordinates.  If I accessed care, my unit leadership might perceive me as less competent.  I feel that others treat me differently if I access care.  My leadership abilities may be called into question if I accessed care.  If I access care, my subordinates and/or my superiors may doubt my competency as a health care provider.  If I accessed care, I would be seen as weak.  I feel that others will discriminate against me if I access care.  If I have to access care, I’m concerned about being perceived as someone who is taking advantage of the system (“faking”).  I would think less of myself as a health care provider if I couldn’t solve my own health problems.  I feel embarrassed when I have to access care. | Each item was scored on a 6-point scale (ranging from 1 = Extremely unlikely to 6 = Extremely likely) then the factor mean was calculated, averaging the scores of all items in the factor. |
| Discomfort accessing care at work (X_2_) | I’m uncomfortable receiving care from my superiors.  I’m uncomfortable receiving care from my subordinates.  I’m uncomfortable receiving care from colleagues.  I’m uncomfortable accessing care where I work.  It makes me uncomfortable to sit in the waiting room with patients while waiting to be seen.  I’m not afforded that same privacy as other patients because my colleagues are part of my circle of care.  Because I access care where I work, when I seek care, people notice or find out I was there. | Each item was scored on a 6-point scale (ranging from 1 = Extremely unlikely to 6 = Extremely likely) then the factor mean was calculated, averaging the scores of all items in the factor. |
| Knowledge and ability to access care (X_3_) | I don’t know how to access the services available to me.  I don’t know what services are available to me.  I’m unfamiliar with any policies that pertain to seeking care in the CAF (e.g., accessing care externally to CAF or switching CDUs).  I find it difficult to navigate the administrative processes necessary to seek some types of care (e.g., mental health, civilian care). | Each item was scored on a 6-point scale (ranging from 1 = Extremely unlikely to 6 = Extremely likely) then the factor mean was calculated, averaging the scores of all items in the factor. |
| Staffing and workload resources (X_4_) | My workload is too heavy for me to leave and access care.  I don’t have time to access care.  I don’t want to leave my colleagues short-staffed to go access care.  I would have difficulty getting time off to access care.  If I need to access care, I am not replaceable. | Each item was scored on a 6-point scale (ranging from 1 = Extremely unlikely to 6 = Extremely likely) then the factor mean was calculated, averaging the scores of all items in the factor. |
| Conflicts with career goals  (X_5_) | If I were going on course, seeking care would prevent me from being able to go.  Accessing care will prevent me from receiving a posting that I want in the future.  Accessing care can result in my being medically released.  If I were being deployed, accessing care would prevent me from deploying.  Accessing care will harm my future chances of promotion.  Accessing care would harm my career. | Each item was scored on a 6-point scale (ranging from 1 = Extremely unlikely to 6 = Extremely likely) then the factor mean was calculated, averaging the scores of all items in the factor. |
| Organizational and social support (X_6_) | My chain of command discourages the use of health services.  My Commanding Officer does not value my health.  My immediate supervisor does not support my accessing health services.  My colleagues (of similar rank) would not support my decision to access health care if needed. | Each item was scored on a 6-point scale (ranging from 1 = Extremely unlikely to 6 = Extremely likely) then the factor mean was calculated, averaging the scores of all items in the factor. |
| Concerns about Privacy (X_6_) | I don’t believe that there is sufficient monitoring for appropriate access of CFHIS files.  When I seek care, my CFHIS file may be seen by those who shouldn’t access it.  When I access care, my colleagues are able to see why I’ve sought care in the past.  I have concerns about the confidentiality of the information I share. | Each item was scored on a 6-point scale (ranging from 1 = Extremely unlikely to 6 = Extremely likely) then the factor mean was calculated, averaging the scores of all items in the factor. |
| Treatment preferences (X_8_) | I want to solve the problem on my own rather than access care.  When I am sick, I think the problem might get better by itself.  I believe that professional care probably will not be more helpful than what I can do myself. | Each item was scored on a 6-point scale (ranging from 1 = Extremely unlikely to 6 = Extremely likely) then the factor mean was calculated, averaging the scores of all items in the factor. |

1. Health related covariate included in the analysis.

| **Construct** | **Survey wording** | **Scoring** |
| --- | --- | --- |
| Intention | When faced with a [*mental health* or *physical health*] issue, I intend to access care. Options included:   1. Strongly Disagree 2. Disagree 3. Somewhat Disagree 4. Neutral 5. Somewhat Agree 6. Agree 7. Strongly Agree | Single item agreement was scored on a 7-point scale (ranging from 1 = Strongly Disagree to 7 = Strongly Agree) |
| Past negative experience | I have had past negative experiences when accessing care. Options included:   1. Strongly Disagree 2. Disagree 3. Somewhat Disagree 4. Somewhat Agree 5. Agree 6. Strongly Agree | Single item agreement was scored on a 6-point scale (ranging from 1 = Strongly Disagree to 6 = Strongly Agree) |
| Perceived health | In general, how would you rate your [*mental health* or *physical health*]? Options included:   1. Very Poor 2. Poor 3. Fair 4. Very Good 5. Excellent | Single item reflecting current health on a 5-point scale |
| Past diagnosis | Have you been diagnosed with a chronic injury or had a serious injury in the past 2 years? Option included:   1. Yes 2. No   Have you been diagnosed with a mental health disorder in the past 2 years? Options included:   1. Yes 2. No | Composite categorical variable coded respondents as having no past diagnoses, having received a physical health diagnosis, having received a mental health diagnosis, or having received both a mental health and physical health diagnoses survey. |
| Past year access to care | How many times in the past year have you sought formal care for an illness? | A dichotomous variable was coded respondents as 1 = accessed care at least once in the last year and 0 = had not accede care in the last year. |

1. Demographic Covariates – Hypothetical access to care using four health scenarios with increasing symptoms severity.

| **Construct** | **Survey wording** | **Modelling** |
| --- | --- | --- |
| Gender | What is your gender?  Options included:   - Male - Female - Or please specify: ___________ - Prefer not to say | Categorical variable |
| Age | What is your age group? Options included:   - 24 years and under - 25-34 years - 35-44 years - 45-54 years - 55-64 years - 65 years and over | Single item agreement was scored on a 6-point scale (ranging from 1 = Strongly Disagree to 6 = Strongly Agree) |
| Trade | What is your current occupation? Options included:   - Biomedical Electronics Technologist - Bioscience Officer - Clerical Staff (HRA) - Clinical Chaplain - Dental Officer - Dental Technician - Healthcare Administrator - Health service Operations - Medical Assistant - Medical Laboratory Technologist - Medical Officer - Medical Officer: Specialty - Medical Radiation Technologist - Medical Technician - Nursing Officer - Operating Room Technician - Pharmacy Officer - Physician Assistant - Physiotherapy Officer - Social Worker - Preventive Medicine Technician - Other: _____ | Composite categorical variable coded respondents as 1. Core clinic (Medical Officer  Medical Technician / Medical Assistant  Nursing Officer  Physician Assistant), 2. Close support (Clinical Chaplain  Health Care Administration Officer  Health Service Operations  Pharmacy Officer  Physiotherapy Officer  Social Work Officer), 3. Dental (Dental Officer  Dental Technician) or 4. Specialty (remaining). |
| Language | Please check all that apply. What is your First Official Language? Options included:   - English - French | Composite categorical variable coded respondents as having no past diagnoses, having received a physical health diagnosis, having received a mental health diagnosis, or having received both a mental health and physical health diagnoses survey. |
| Location | What location are you currently in: (We are asking this information because we know there are differences in health care accessibility depending on the size and location of your base [e.g., rural vs. suburban]). Options included:  British Columbia:  • Comox  • Esquimalt  • Vancouver  • Victoria  • Other:___________  Alberta:  • Calgary  • Cold Lake  • Edmonton  • Suffield  • Wainwright  • Other:___________  Manitoba:  • Shilo  • Moose Jaw  • Winnipeg  • Other:___________  Saskatchewan:  • Dundurn  • Moose Jaw  • Regina  • Saskatoon  • Other:___________  Ontario:  • Borden  • Hamilton  • Kingston  • North Bay  • Ottawa  • Petawawa  • Sudbury  • Thunder Bay  • Toronto  • Trenton  • Other:___________  Quebec:  • Bagotville  • Montreal  • Rimouski  • Sherbrooke  • Trois-Rivières  • Valcartier  • Other:___________  New Brunswick:  • Gagetown  • Other:___________  Newfoundland and Labrador:  • Goose Bay  • Gander  • St. John’s  • Other:___________  Nova Scotia:  • Cape Breton  • Greenwood  • Halifax  • Shearwater  • Other:___________  Territories:  • Yellowknife  • Other:___________ | Composite categorical variable coded respondents location as rural remote, semi-rural, peri-urban, and urban using methods detailed in Richer, I., Thériault, F., Strauss, B., & Gauthier, M. (2017). Correlates of risky drinking among Regular Canadian Armed Forces members. Unpublished research protocol. Of note, because of low cell counts, rural remote and semi-rural were collapsed into a single rural category during analysis. |
