# Supplementary material for: Direct and indirect barriers to hypothetical access to care among Canadian forces health services personnel
Source: Res Health Serv Reg. 2023 Aug 2;2:11. doi: 10.1007/s43999-023-00026-6 (PMC11281741; doi:10.1007/s43999-023-00026-6)
Supplement: Supplementary file 3 — Additional file 3. [file 43999_2023_26_MOESM3_ESM.docx]

**Online supplement 3 – Process Models**

Table 3.1.

*Full direct and indirect regression results for back injury*

| Component | Factor | Direct Effect | | | | | Indirect Effect | | |
| --- | --- | --- | --- | --- | --- | --- | --- | --- | --- |
|  |  |  | | | 95% C.I. | |  | 95% C.I. | |
|  |  | *B(se)* | *Z* | *p* | *LL* | *UL* | *B(se)* | *LL* | *UL* |
| Capability | Knowledge and ability to access care (X_3_) | .01(.13) | 0.09 | .93 | -.25 | .27 | -.02(.03) | -.09 | .04 |
| Opportunity | Staffing and workload resources (X_4_) | **-.50(.15)** | **-3.39** | **.001** | **-.78** | **-.21** | -.04(.03) | -.11 | .02 |
|  | Organizational and social support (X_6_) | -.22(.17) | -1.34 | .18 | -.56 | .10 | .03(.04) | -.05 | 1.11 |
| Motivation | CFHS personnel identity (X_1_) | .38(.21) | 1.79 | .07 | -.04 | .80 | -.08(.05) | -.18 | .01 |
|  | Discomfort accessing care at work (X_2_) | -.04(.17) | -0.21 | .83 | -.36 | .29 | .04(.03) | -.03 | .10 |
|  | Conflicts with career goals (X_5_) | -.01(.16) | -0.04 | .97 | -.32 | .30 | **-.08(.04)** | **-.17** | **-.02** |
|  | Treatment preferences (X_8_) | -.05(.17) | -0.28 | 78 | -.38 | .29 | -.03(.03) | -.11 | .03 |
|  | Concerns about privacy (X_7_) | -.19(.15) | -1.24 | .22 | -.50 | .11 | .02(.03) | -.03 | .09 |
|  | **Intention** | **.33(.09)** | **3.78** | **.002** | **.16** | **.50** |  |  |  |
|  | Past negative experience | -.04(.33) | -0.12 | .90 | -.68 | .60 |  |  |  |
|  | Perceived physical health | .28(.18) | 1.57 | .12 | -.07 | .62 |  |  |  |
|  | **Past year access to care** | **.88(.36)** | **2.44** | **.01** | **.17** | **1.58** |  |  |  |

Table 3.2.

*Full direct and indirect regression results for pneumonia*

| System | Factor | Direct Effect | | | | | Indirect Effect | | |
| --- | --- | --- | --- | --- | --- | --- | --- | --- | --- |
|  |  |  | | | 95% C.I. | |  | 95% C.I. | |
|  |  | *B(se)* | *Z* | *p* | *LL* | *UL* | *B(se)* | *LL* | *UL* |
| Capability | Knowledge and ability to access care (X_3_) | **-.30(.13)** | **-2.30** | **.02** | **-.55** | **-.04** | -.02(.03) | -.08 | .04 |
| Opportunity | Staffing and workload resources (X_4_) | **-.37(.14)** | **-2.72** | **.001** | **-.63** | **-.10** | -.05(.03) | -.13 | .004 |
|  | Organizational and social support (X_6_) | .09(.17) | 0.52 | .60 | -.25 | .42 | .02(.04) | -.06 | .11 |
| Motivation | CFHS personnel identity (X_1_) | .11(.20) | 0.55 | .58 | -.28 | .50 | -.07(.05) | -.18 | .02 |
|  | Discomfort accessing care at work (X_2_) | -.15(.15) | -0.98 | .33 | -.45 | .15 | .03(.03) | -.03 | .10 |
|  | Conflicts with career goals (X_5_) | -.04(.15) | -0.26 | .80 | -.33 | .25 | **-.07(.04)** | **-.15** | **-.01** |
|  | Treatment preferences (X_8_) | -.19(.16) | -1.19 | .24 | -.51 | .13 | -.01(.03) | -.09 | .04 |
|  | Concerns about privacy (X_7_) | .28(.15) | 1.82 | .07 | -.02 | .58 | .01(.03) | -.05 | .08 |
|  | **Intention** | **.27(06)** | **4.85** | **<.001** | **.16** | **.38** |  |  |  |
|  | Past negative experience | -.10(.30) | -0.34 | .73 | -.69 | .48 |  |  |  |
|  | **Perceived physical health** | **.31(.10)** | **2.97** | **.003** | **.10** | **.51** |  |  |  |
|  | Past year access to care | .56(.35) | 1.60 | .11 | -.12 | 1.24 |  |  |  |

Table 3.3.

*Full direct and indirect regression results for depression*

| System | Factor | Direct Effect | | | | | Indirect Effect | | |
| --- | --- | --- | --- | --- | --- | --- | --- | --- | --- |
|  |  |  | | | 95% C.I. | |  | 95% C.I. | |
|  |  | *B(se)* | *Z* | *p* | *LL* | *UL* | *B(se)* | *LL* | *UL* |
| Capability | Knowledge and ability to access care | **-.37(.14)** | **-2.60** | **.01** | **-.67** | **-.09** | -.04(.03) | -.12 | .01 |
| Opportunity | Staffing and workload resources | -.20(.13) | -1.47 | .14 | -.46 | .07 | .02(.03) | -.03 | .08 |
|  | Organizational and social support | -.01(.17) | -0.06 | .96 | -.34 | .32 | .01(.04) | -.07 | .10 |
| Motivation | CFHS personnel identity | .28(.19) | 1.41 | .16 | -.11 | .66 | -.01(.05) | -.13 | .08 |
|  | Discomfort accessing care at work | -.13(.15) | -0.86 | .39 | -.43 | .17 | -.01(.03) | -.07 | .05 |
|  | Conflicts with career goals | **-.35(.15)** | **-2.28** | **.02** | **-.64** | **-.05** | **-.08(.04)** | **-.18** | **-.01** |
|  | Treatment preferences | **-.47(.17)** | **-2.74** | **.006** | **-.80** | **-.13** | **-.08(.05)** | **-.19** | **-.01** |
|  | Concerns about privacy | .12(.16) | 0.71 | .47 | -.20 | .44 | .02(.04) | -.05 | .09 |
|  | **Intention** | **.30(.08)** | **3.61** | **.0003** | **.14** | **.46** |  |  |  |
|  | Negative past experiences | .15(.35) | 0.42 | .68 | -.54 | .84 |  |  |  |

Table 3.4

*Full direct and indirect regression results for PTSD*

| System | Factor | Direct Effect | | | | | Indirect Effect | | |
| --- | --- | --- | --- | --- | --- | --- | --- | --- | --- |
|  |  |  | | | 95% C.I. | |  | 95% C.I. | |
|  |  | *B(se)* | *Z* | *p* | *LL* | *UL* | *B(se)* | *LL* | *UL* |
| Capability | Knowledge and ability to access care | .01(.13) | 0.10 | .92 | -.24 | .26 | -.03(.03) | -.09 | .02 |
| Opportunity | Staffing and workload resources | -.10(.14) | -0.74 | .46 | -.38 | .17 | .01(.03) | -.04 | .08 |
|  | Organizational and social support | -.19(.16) | -1.25 | .21 | -.50 | .11 | .01(.04) | -.07 | .09 |
| Motivation | CFHS personnel identity | .16(.20) | 0.81 | .42 | -.23 | .55 | -.02(.05) | -.12 | .07 |
|  | Discomfort accessing care at work | .18(.16) | 1.08 | .28 | -.14 | .49 | .01(.03) | -.05 | .07 |
|  | Conflicts with career goals | **-.46(.17)** | **-2.77** | **.006** | **-.79** | **-.14** | **-.08(.04)** | **-.18** | **-.01** |
|  | Treatment preferences | **-.52(.18)** | **-2.81** | **.005** | **-.88** | **.16** | **-.10(.05)** | **-.21** | **-.03** |
|  | Concerns about privacy | -.13(.16) | -0.85 | .40 | -.45 | .18 | .01(.03) | -.05 | .08 |
|  | **Intention** | **.29(.08)** | **3.43** | **.001** | **.12** | **.45** |  |  |  |
|  | Negative past experiences | -.19(.35) | -0.53 | .59 | -.86 | .49 |  |  |  |
|  | Past year access to care | -.22(.20) | -1.11 | .27 | -.61 | .17 |  |  |  |
